# Abrogation of CC Chemokine Receptor 9 Ameliorates Ventricular Electrical Remodeling in Mice After Myocardial Infarction

**DOI:** 10.3389/fcvm.2021.716219

**Published:** 2021-10-12

**Authors:** Yan Huang, Hua-Sheng Ding, Tao Song, Yu-Ting Chen, Teng Wang, Yan-Hong Tang, Hector Barajas-Martinez, Cong-Xin Huang, Dan Hu

**Affiliations:** ^1^Department of Cardiology, Renmin Hospital of Wuhan University, Wuhan, China; ^2^Cardiovascular Research Institute, Wuhan University, Wuhan, China; ^3^Hubei Key Laboratory of Cardiology, Wuhan, China; ^4^Lankenau Institute for Medical Research, Lankenau Heart Institute, Wynnwood, PA, United States; ^5^Jefferson Medical College, Philadelphia, PA, United States

**Keywords:** myocardial infarction, chemokine receptor 9, action potential, ion channel, calcium transient, cardiac conduction, connexin 43

## Abstract

**Introduction:** Myocardial infarction (MI) triggers structural and electrical remodeling. CC chemokine receptor 9 (CCR9) mediates chemotaxis of inflammatory cells in MI. In our previous study, CCR9 knockout has been found to improve structural remodeling after MI. Here, we further investigate the potential influence of CCR9 on electrical remodeling following MI in order to explore potential new measures to improve the prognosis of MI.

**Methods and Results:** Mice was used and divided into four groups: CCR9^+/+^/Sham, CCR9^−/−^/Sham, CCR9^+/+^/MI, CCR9^−/−^/MI. Animals were used at 1 week after MI surgery. Cardiomyocytes in the infracted border zone were acutely dissociated and the whole-cell patch clamp was used to record action potential duration (APD), L-type calcium current (*I*_*Ca,L*_) and transient outward potassium current (*I*_*to*_). Calcium transient and sarcoplasmic reticulum (SR) calcium content under stimulation of Caffeine were measured in isolated cardiomyocytes by confocal microscopy. Multielectrode array (MEA) was used to measure the conduction of the left ventricle. The western-blot was performed for the expression level of connexin 43. We observed prolonged APD_90_, increased *I*_*Ca,L*_ and decreased *I*_*to*_ following MI, while CCR9 knockout attenuated these changes (APD_90_: 50.57 ± 6.51 ms in CCR9^−/−^/MI vs. 76.53 ± 5.98 ms in CCR9^+/+^/MI, *p* < 0.05; *I*_*Ca,L*_: −13.15 ± 0.86 pA/pF in CCR9^−/−^/MI group vs. −17.05 ± 1.11 pA/pF in CCR9^+/+^/MI, *p* < 0.05; *I*_*to*_: 4.01 ± 0.17 pA/pF in CCR9^−/−^/MI group vs. 2.71 ± 0.16 pA/pF in CCR9^+/+^/MI, *p* < 0.05). The confocal microscopy results revealed CCR9 knockout reversed the calcium transient and calcium content reduction in sarcoplasmic reticulum following MI. MEA measurements showed improved conduction velocity in CCR9^−/−^/MI mice (290.1 ± 34.47 cm/s in CCR9^−/−^/MI group vs. 113.2 ± 14.4 cm/s in CCR9^+/+^/MI group, *p* < 0.05). Western-blot results suggested connexin 43 expression was lowered after MI while CCR9 knockout improved its expression.

**Conclusion:** This study shows CCR9 knockout prevents the electrical remodeling by normalizing ion currents, the calcium homeostasis, and the gap junction to maintain APD and the conduction function. It suggests CCR9 is a promising therapeutic target for MI-induced arrhythmia, which warrants further investigation.

## Introduction

Myocardial infarction (MI) is a disease of continuous ischemia and necrosis of cardiomyocytes caused by thrombus formation due to coronary atherosclerosis and rupture of vascular plaques, resulting in acute vascular occlusion (1). It brings a huge burden to the national economy and seriously affects the quality of life of patients. It is estimated that globally, ischemic heart disease affects around 126 million individuals (1,655 per 100,000), which is approximately 1.72% of the world's population. Nine million deaths were caused by IHD globally. By the year 2030, the prevalence rate is expected to exceed 1,845 per 100,000 population. The cost of MI is expected to account for 1–1.5% of the gross domestic product in countries like the United States (2). Cardiac remodeling including structural and electrical remodeling occurs after MI, which lead to hypertrophy, heart failure, arrhythmias as well as sudden cardiac death (SCD) (3). And ventricular arrhythmia is the leading cause responsible for SCD after MI (4). Electrical remodeling, including changes in ion channel currents and action potentials (APs) by myocardial conduction disorder, is pathological basis of arrhythmias following MI. The changes of repolarization currents, such as reduction in potassium current, including the transient outward current (*I*_*to*_), the inward rectifier potassium current (*I*_K1_), as well as the slow delayed rectifier potassium current (*I*_*Ks*_), could lead to the prolongation of ventricular action potential duration (APD), and increased dispersion of ventricular repolarization, which are potential mechanisms for ventricular arrhythmias and SCD (5). As far, targeted drugs and various interventional techniques are the main clinical treatments to improve patient outcomes following MI. However, potential adverse reactions and poor prognosis cannot be totally resolved (6–8). Therefore, noninvasive, nonpharmacological, and clinically applicable technical approaches are being explored, such as stem and progenitor cell therapy or genetic therapy, since these promising strategies are specifically targeted and without drug toxicity compared with traditional means (5, 9, 10).

Chemokine receptors are mainly expressed on inflammatory cells like neutrophils, monocytes and macrophages. They also exist on other cells including endothelial cells and tumor cells (11). Chemokines as well as chemokine receptors play an important role in cardiovascular diseases: chemokine receptor 2 (CCR2)/chemokine ligand 2 (CCL2) participate in atherosclerosis and pathophysiology in MI. Inhibition of CCL2/CCR2 by using inhibitors or genetic technology can reduce inflammation and inhibit detrimental ventricular remodeling (12). Chemokine ligand 21 (CCL21)/chemokine receptor 7 (CCR7) play an important role in MI, neutralization antibody of CCL21 can improve ventricular remodeling by reducing infarct size and suppressing collagen content in myocardium after acute MI (13). Development of atherosclerosis needs the chemokine receptor 1 (CCR1)/chemokine receptor 5 (CCR5) to recruit monocytes (14). Thymus-expressed chemokine (CCL25) is the only known ligand for CC chemokine receptor 9 (CCR9). Like other chemokine receptors, CCR9 is also detected on lymphocytes, monocytes, macrophages and dendritic cells (DCs) (15). CCR9 was found to be involved in inflammatory diseases like inflammatory bowel disease, rheumatoid arthritis, and cancer (16–20). In cardiovascular system, only 2 literatures reported the relationship between CCR9 and heart diseases (21, 22). In the previous study, we found CCR9 abrogation could improve ventricular structural remodeling by reducing infract size, inhibiting inflammation and fibrosis after MI, thus first reported CCR9 plays a crucial role in MI (21). In the other study, CCR9 was found to be related to pathological myocardial hypertrophy (22). Our present study is aimed to use CCR9 gene knockout mice to explore the effect of CCR9 in ventricular electrical remodeling following MI, and to discover novel methods that can prevent deteriorating outcomes after MI.

## Materials and Methods

### Experimental Mice

Global CCR9 knockout mice (CCR9-KO, C57BL/6J background) were purchased from the European Mouse Mutant Archive (EM:02293. B6;129-Ccr9tm1Dgen/H). The mice were bred and kept in our animal house with specific pathogen-free environment in Renmin hospital of Wuhan university. Male mice with an age of 6–8 weeks (body weight 24–27g) were used in our study. All the protocols in our experiment were authorized by the Animal Care and Use Committee of Renmin Hospital of Wuhan University. The pain and suffering of animals were reduced to minimize during the experiments.

### Left Coronary Artery Ligation

The surgery was conducted as described before (21). In short, mice were anesthetized with sodium pentobarbital (intraperitoneal injection, 50 mg/kg). After getting anesthesia, small animal ventilator was used to maintain normal breathing. For subjects in the MI groups, the chest was opened, the heart was fully exposed, and the left anterior descending coronary was ligated. The mice in sham groups underwent the pericardium opening as well but without ligation. The mice were divided into four group: wild type sham group (CCR9^+/+^/Sham), CCR9 knockout sham group (CCR9^−/−^/Sham), wild type MI group (CCR9^+/+^/MI), and CCR9 knockout MI group (CCR9^−/−^/MI). The animals were used for experiments at 1 week following MI surgery.

### Isolation of Single Ventricular Cardiomyocytes

After the mice were anesthetized by sodium pentobarbital, the heart was quickly removed as soon as the chest was opened, then soaked in the iced Ca^2+^-free Tyrode's solution and appendages were pruned. The ascending aorta cannulation was quickly performed and perfused the heart with Ca^2+^-free Tyrode's solution for around 5 mins, followed by enzymatic solution for 6–7 mins until the full digestion was achieved. The heart was then transferred to Ca^2+^-free Tyrode's solution containing BSA (1 mg/ml) to stop digestion. Then, the left ventricle was cut out and the infarct border zone was reserved and dispersed into single cell with a polished Pasteur pipette. The cells were kept in the BSA solution at room temperature after the cell suspension was filtered.

### Patch Clamp Recording

Since APD change in the perfused heart was observed in both CCR9^+/+^/MI mice and CCR9^−/−^/MI mice in our previous study, we wondered whether there were cardiac ionic currents alternation in single cardiomyocytes. Patch-clamp analyses were performed using acutely isolated ventricular myocytes from the infarcted border zone. Action potential duration (APD), transient outward potassium current (*I*_*to*_) and L-type calcium current (*I*_*Ca,L*_) were recorded by whole-cell patch-clamp. The glass microelectrodes were pulled and the microelectrode resistance ranges from 1 to 3 MΩ. A gentle negative pressure was given to rupture the cell membrane. The pipette solution for AP recording was (mmol/L): NaCl 5; KCl 15; K-glutamate 130; MgCl2 21; MgATP 5; CaCl2 1; HEPES 10; EGTA 5, and Tyrode's solution was used as the extracellular solution. *I*_*Ca,L*_ was measured with bath solution (mmol/L: CsCl 20; TEA-Cl 135; MgCl2 0.5; HEPES 5; CaCl2 1.8) and pipette solution (mmol/L: CsOH 110; CsCl 20; TEACl 10; Aspartate 90; HEPES 10; EGTA 10; MgATP 5; creatine phosphate sodium 5; GTP 0.4; leupeptin 0.1). For *I*_*Ca,L*_ recording, a slow voltage ramp with holding potential ranging from −70 to −45 mV was applied to inactivate sodium and T-type calcium current, and then voltage was increased to +65 mV step by step in 10 mV increments. The bath solution for *I*_*to*_ was (mmol/L): NaCl 138; MgCl2 1.0; KCl 5.4; CaCl2 1.8; HEPES 10; Glucose 10; nifedipine 0.02 and pipette solution (mmol/L) was: KOH 130; KCl 15; MgATP 5; CaCl2 1; L-glutamic acid 130; MgCl2 1; NaCl 5; EGTA 5; HEPES 10. *I*_*to*_ was measured with a holding potential −80 mV and using a depolarizing voltage step to +70 mV from a 40 ms pre-pulse of −40 mV to inactivate the sodium channel.

### Calcium Image

Isolated ventricular myocytes from infarct border zone were centrifuged (1,200 rpm, 3 min). The supernatant was removed, and the precipitation was resuspended in Tyrode's solution in a 1.5 ml centrifuge tube. Then 10 μM Fluo 4-AM was used to incubate the cells without light for 1 h at 37°C. After incubation, the cells were washed by using Tyrode's solution for three times. Then, cells were kept on the ice and calcium transients were measured using a dual-beam excitation fluorescence photometry setup (Leica, Wetzlar, Germany). The emission wavelength and receiving wavelength for recording was 460–480 nm and 500–550 nm, respectively. A home-made cell groove with two platinum wires at one end was used to pass the electrical stimulation to the cells. The cells were given a field stimulation, of which the stimulus frequency was 0.5 and 1 Hz. The stimulation was repeated six times. After recording Ca^2+^ transient in both stimulation frequency, we switched the perfusion to normal Tyrode's solution with 10 mM caffeine to empty Ca^2+^ storage in sarcoplasmic reticulum (SR) to record the SR calcium content.

### Multielectrode Array (MEA)

MEA measurements were made *in vitro* mouse heart 1 week after MI. The preparation of the isolated perfused heart was as above. The isolated heart was perfused with 37°C Tyrode's solution. After recovery of the spontaneous rhythms, the MEA dish containing 32 monopolar electrodes (32 Map) with interelectrode distance of 500 μm to cover a 3 × 3 mm square was placed on left ventricle. Electrical stimulation with the stimulating voltage ranging from 1V to 7V and a duration of 1 ms was applied. Cardio2D+ software (Multi Channel Systems) was used to acquire the data sampled at 10 kHz. Activation maps were produced and the conduction velocity (CV) was calculated by analyzing the whole data.

### Western Blot

Structural remodeling occurs after MI, which can lead to changes in gap junctions among cardiac myocytes. In the previous study, we observed CCR9 knockout could improve these serious structural remodeling, so we wondered whether CCR9 knockout could affect these changes in gap junction. Here, western blot was applied to detect the expression level of connexin 43 (Cx43) in ventricular tissue from infracted border zone. The mice were sacrificed to dissect the LV infract border zone. The dissected tissues were grinned to extracted proteins and quantified protein concentrations by BCA assay kit. Forty microgram protein sample was loaded and proteins were separated by 10% sodium dodecylsulphate (SDS)-polyacrylamide gel electrophoresis (PAGE), then transferred to a polyvinylidene difluoride (PVDF) membrane. After the transferring, the PVDF membrane was removed and transferred into sealing solution for mild vibration at 4°C overnight with anti-Cx43 primary antibody (1:1,000, abcam, ab235585) followed by rabbit IgG (1:2,000, abcam, ab6721) for 1 h at room temperature. Development and photographic recording were performed with Bio-RDA gel imaging system.

### Statistical Analysis

All data were presented as the means ± SEM. Unpaired student's *t*-test or one-way analysis of variance (ANOVA) was used to make comparisons between two groups by using graphPad Prism version 5. Chi-Square Test with Fisher's exact test was used for the categorical variables. *P* ≤ 0.05 was considered significant statistically.

## Results

### CCR9 Knock Down Inhibits APD Prolongation Following MI

**Figure 1:**
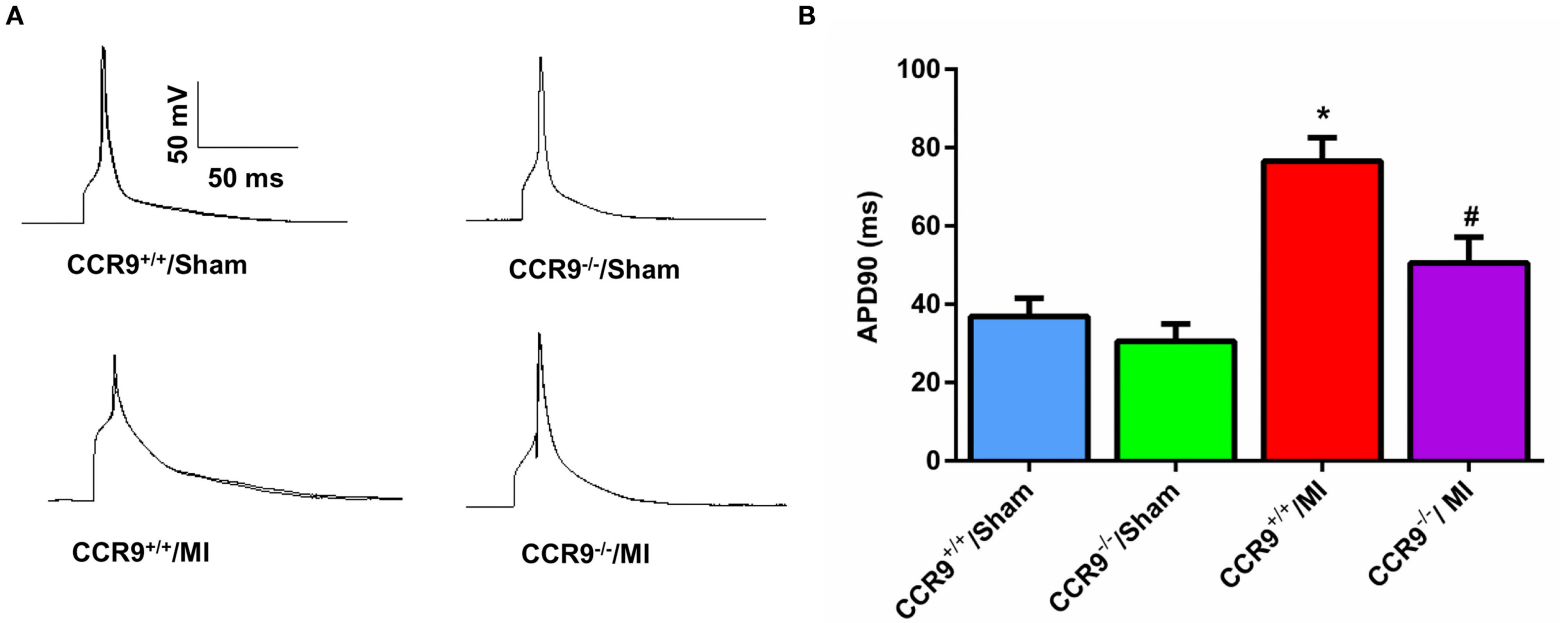
CCR9 knock down inhibits APD prolongation following MI. **(A)** Representive action potential recordings in mouse ventricular myocytes from each group. **(B)** Quantified analysis of APD_90_ in CCR9^+/+^ and CCR9^−/−^ mice with sham operation or MI surgery at 1 week (*p < 0.05 vs. CCR9^+/+^/Sham, ^#^*p* < 0.05 vs. CCR9^+/+^/MI).

AP was recorded in isolated left ventricular cardiomyocytes around the infraction area using the whole cell patch clamp. MI markly prolonged the APD_90_ (76.53 ± 5.98 ms in CCR9^+/+^/MI, *n* = 6 cells from 3 hearts, vs. 36.91 ± 4.64 ms in CCR9^+/+^/Sham, *n* = 11 cells from 3 hearts, *p* < 0.05). Loss of CCR9 inhibited the plongation of APD induced by MI (50.57 ± 6.51 ms in CCR9^−/−^/MI, *n* = 6 cells from 3 hearts vs. 76.53 ± 5.98 ms in CCR9^+/+^/MI, *n* = 6 cells from 3 hearts, *p* < 0.05; Figure 1).

### CCR9 Knockout Attenuates MI-Induced Calcium Current Increase and Reduction of Transient Outward Potassium Current After MI

**Figure 2:**
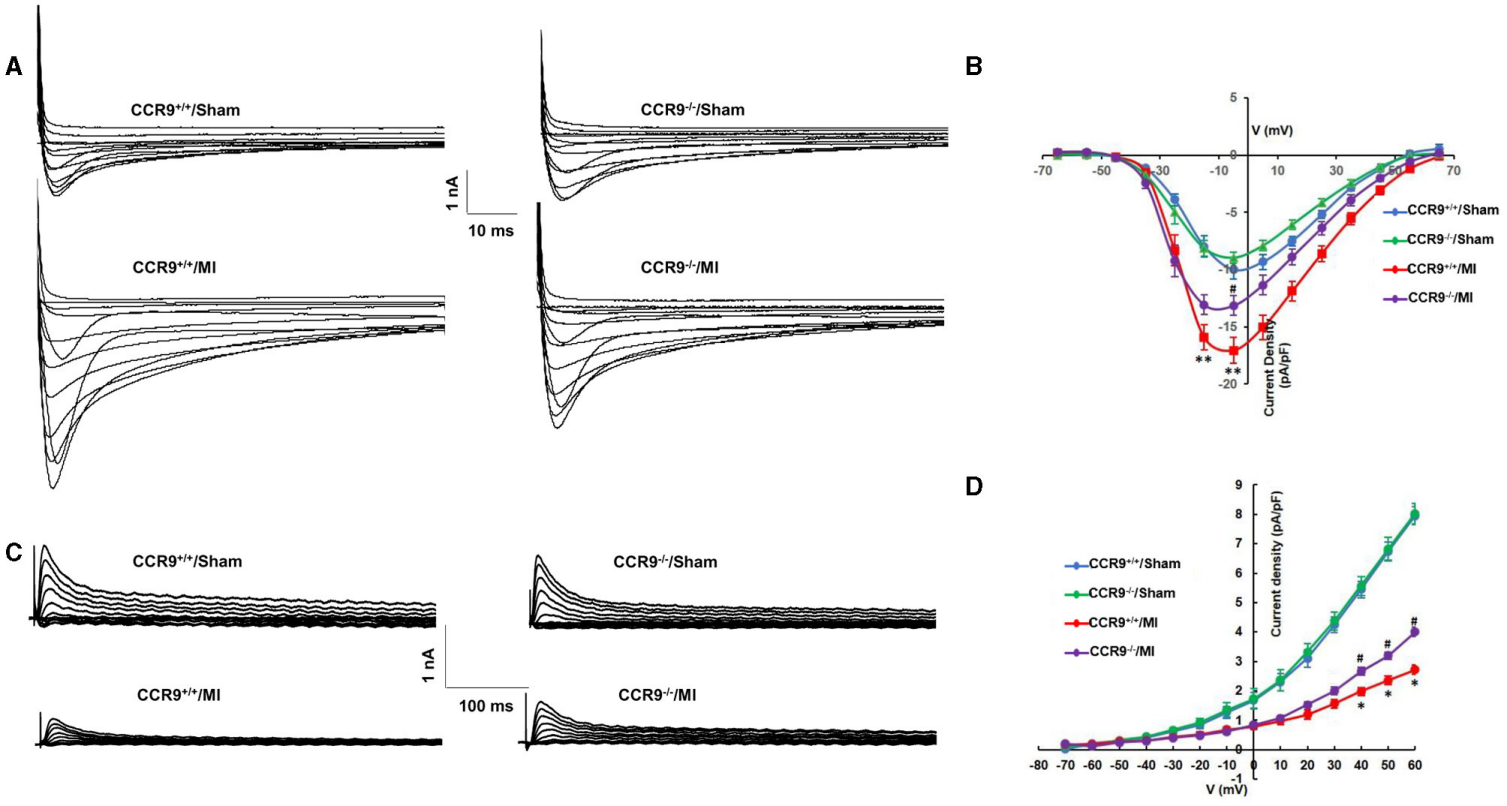
CCR9 knockoutattenuates MI-induced calcium current increasing and reduction of transient outward potassium current after MI. **(A)** Representive raw traces of *I*_*Ca,L*_ in mouse ventricular myocytes from each group. **(B)** Normalized current–voltage relationship for *I*_*Ca,L*_ (**p < 0.01 vs. CCR9^+/+^/Sham, ^#^*p* < 0.05 vs. CCR9^+/+^/MI). **(C)** Representive raw traces of *I*_*to*_ in ventricular myocytes from each group. **(D)** Normalized current–voltage relationship for *I*_*to*_ (*p < 0.05 vs. CCR9^+/+^/Sham, ^#^*p* < 0.05 vs. CCR9^+/+^/MI).

The original *I*_*Ca,L*_ traces for isolated ventricular myocytes from the infarcted border zone among different groups were shown in Figure 2A. MI markedly increased the amplitude of *I*_*Ca,L*_, which can be reduced by CCR9 knockout. The current density-voltage (*I*-*V*) correlations for the *I*_*Ca,L*_ of each group were shown in Figure 2B, which showed that MI significantly increased the peak current when compared with the WT groups (−17.05 ± 1.11 pA/pF in CCR9^+/+^/MI group, *n* = 8 cells from 4 hearts vs. −10.02 ± 0.83 pA/pF in CCR9^+/+^/Sham, *n* = 9 cells from 3 hearts, *p* < 0.01; −13.15 ± 0.86 pA/pF in CCR9^−/−^/MI group, *n* = 10 cells from 4 hearts vs. −8.76 ± 0.49 pA/pF in CCR9^−/−^/Sham, *n* = 9 cells from 3 hearts, *p* < 0.01), while CCR9 knockout reduced the current density compared with CCR9^+/+^/MI (−13.15 ± 0.86 pA/pF in CCR9^−/−^/MI group, *n* = 10 cells from 4 hearts vs. −17.05 ± 1.11 pA/pF in CCR9^+/+^/MI, *n* = 8 cells from 4 hearts, *p* < 0.05). Besides, we also recorded *I*_*to*_, the results showed the peak *I*_*to*_ was significantly reduced in MI mice (+60 mV: 2.71 ± 0.16 pA/pF in CCR9^+/+^/MI group, *n* = 8 cells from 4 hearts vs. 7.95 ± 0.31 pA/pF in CCR9^+/+^/Sham, *n* = 8 cells from 3 hearts, *p* < 0.05; 4.01 ± 0.17 pA/pF in CCR9^−/−^/MI group, *n* = 8 cells from 4 hearts vs. 8.03 ± 0.35 pA/pF in CCR9^−/−^/Sham, *n* = 6 cells from 3 hearts, *p* < 0.05), while CCR9 deficiency notably attenuated MI-induced *I*_*to*_ reduction (4.01 ± 0.17 pA/pF in CCR9^−/−^/MI group, *n* = 8 cells from 4 hearts vs. 2.71 ± 0.16 pA/pF in CCR9^+/+^/MI, *n* = 8 cells from 4 hearts, *p* < 0.05, Figures 2C,D). These data showed the MI induced *I*_*Ca,L*_ increasing and *I*_*to*_ decreasing, while absence of CCR9 can inhibit these ionic currents changing, therefore prevented APD prolongation following MI.

### CCR9 Deficiency Restores Calcium Handling After MI

**Figure 3:**
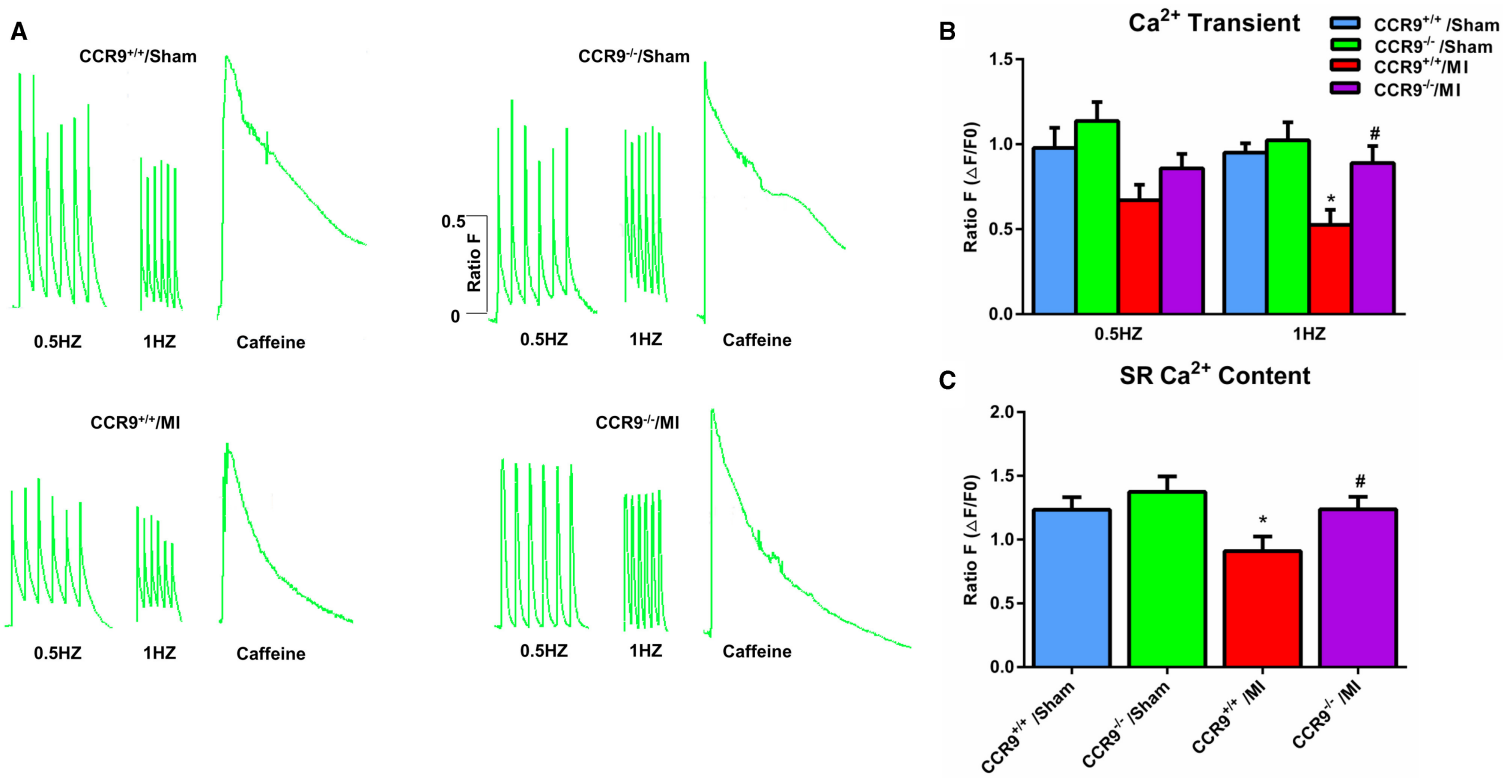
CCR9 deficiency restores calcium handling after MI. **(A)** Representive Ca^2+^transients and SR Ca^2+^content recordings at 0.5 and 1-Hz field stimulation in mouse myocytes from each group. **(B)** Statistical analysis of Ca^2+^transient amplitude (Δ*F*/*F*0) in each groups (*p < 0.05 vs. CCR9^+/+^/Sham, ^#^*p* < 0.05 vs. CCR9^+/+^/MI). **(C)** Quantified analysis of SR Ca^2+^content following 10 mM caffeine perfusion in different groups (*p < 0.05 vs. CCR9^+/+^/Sham, ^#^*p* < 0.05 vs. CCR9^+/+^/MI).

Arrhythmia following MI is associated with calcium homeostasis disorder. We further carried out the calcium image experiment to observe calcium handling in the ventricular cardiomycytes following MI. As shown in Figures 3A,B, MI induced the Ca^2+^ transient amplitudes decreased in both 0.5 and 1 Hz, but was only statistically significant in 1 Hz (0.53 ± 0.09 in CCR9^+/+^/MI group, *n* = 7 cells from 4 hearts, vs. 0.95 ± 0.05 in CCR9^+/+^/Sham group, *n* = 9 cells from 3 hearts, *p* < 0.05; 0.86 ± 0.08 in CCR9^−/−^/MI group, *n* = 13 cells from 4 hearts vs. 1.14 ± 0.11 in CCR9^−/−^/Sham group, *n* = 12 cells from 3 hearts, *p* > 0.05). Loss of CCR9 could restore calcium transient amplitude in 1 Hz (0.86 ± 0.08 in CCR9^−/−^/MI group, *n* = 13 cells from 4 hearts vs. 0.53 ± 0.09 in CCR9^+/+^/MI group, *n* = 7 cells from 4 hearts, *p* < 0.05). No difference was observed in Ca^2+^ transient amplitudes between 0.5 and 1 Hz. The SR calcium content experiment revealed decreased SR Ca^2+^content after MI in CCR9^+/+^/MI mice but not the CCR9^−/−^/MI mice (0.90 ± 0.11 in CCR9^+/+^/MI group, *n* = 8 cells from 4 hearts vs. 1.23 ± 0.10 in CCR9^+/+^/Sham group, *n* = 13 cells from 3 hearts, *p* = 0.05; 1.24 ± 0.09 in CCR9^−/−^/MI group, *n* = 11 cells from 4 hearts vs. 1.37 ± 0.12 in CCR9^−/−^/Sham group, *n* = 11 cells from 3 hearts, *p* > 0.05). CCR9 deficiency restored SR calcium content (1.24 ± 0.09 in CCR9^−/−^/MI group, *n* = 11 cells from 4 hearts vs. 0.90 ± 0.11 in CCR9^+/+^/MI group, *n* = 8 cells from 4 hearts, *p* < 0.05, Figure 3C). On the whole, these results indicate that CCR9 deficiency restores abnormal SR Ca^2+^ storage and Ca^2+^ release caused by MI.

### CCR9 Knockout Improves the Conduction Function After MI

**Figure 4:**
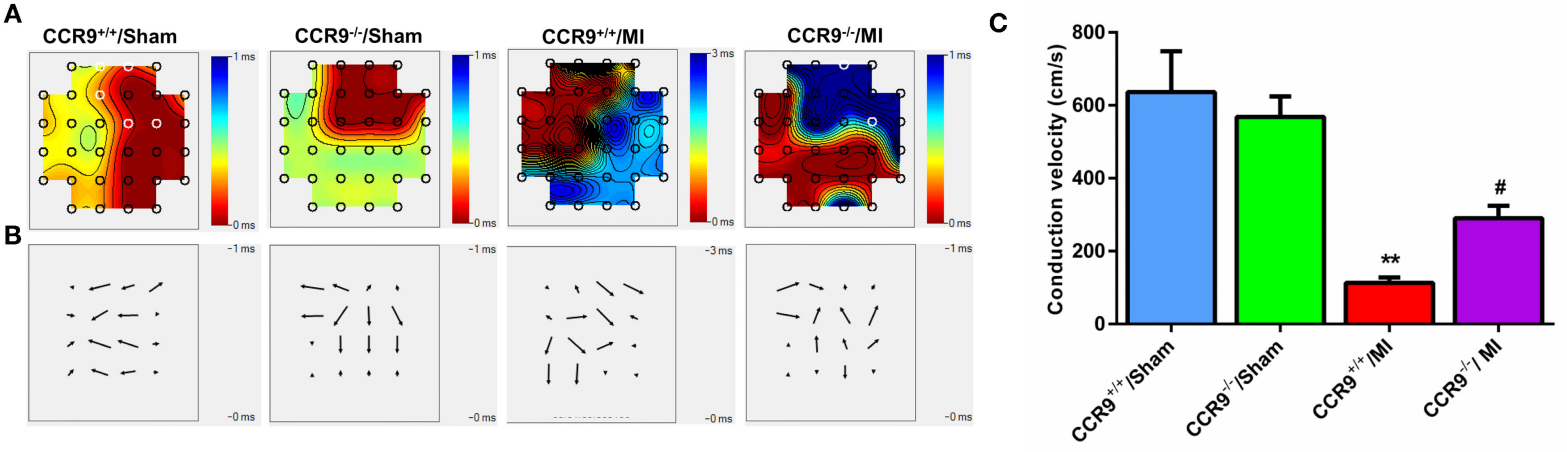
CCR9 knockout improves the conduction function after MI. **(A)** The conduction maps of excitation in the left ventricle recorded by MEA. MI mice presented crowding isochronal, of which the crowding degree was less in the CCR9^−/−^ MI group. **(B)** Heterogeneity of conduction in mice left ventricle. There was more heterogeneous in CCR9^+/+^/MI mice than CCR9^−/−^/MI mice. **(C)** Statistical analysis of CV in each group. CCR9 knockout increased the ventricular CV following MI. (**p < 0.01 vs. CCR9^+/+^/Sham, ^#^*p* < 0.05 vs. CCR9^+/+^/MI).

Conduction activation maps (Figure 4A) as well as CV maps (Figure 4B) on anterior wall of left ventricle surface diaplayed more crowded isochrones and a slower CV in the MI group than that in the sham group (113.2 ± 14.4 cm/s in CCR9^+/+^/MI group, *n* = 5 hearts vs. 636.2 ± 112.1 cm/s in CCR9^+/+^/sham group, *n* = 5 hearts, *p* < 0.01; 290.1 ± 34.47 cm/s in CCR9^−/−^/MI group, *n* = 6 hearts vs. 568.6 ± 56.4 cm/s in CCR9^−/−^/Sham group, *n* = 4 hearts, *p* < 0.01). What's more, left ventricle CV was faster and exhibited better homogeneity in the CCR9^−/−^/MI group (290.1 ± 34.47 cm/s in CCR9^−/−^/MI group, n=6 hearts vs. 113.2 ± 14.4 cm/s in CCR9^+/+^/MI group, *n* = 5 hearts, *p* < 0.05; Figure 4C).

### CCR9 Knock Out Preserves the Expression of Cx43

**Figure 5:**
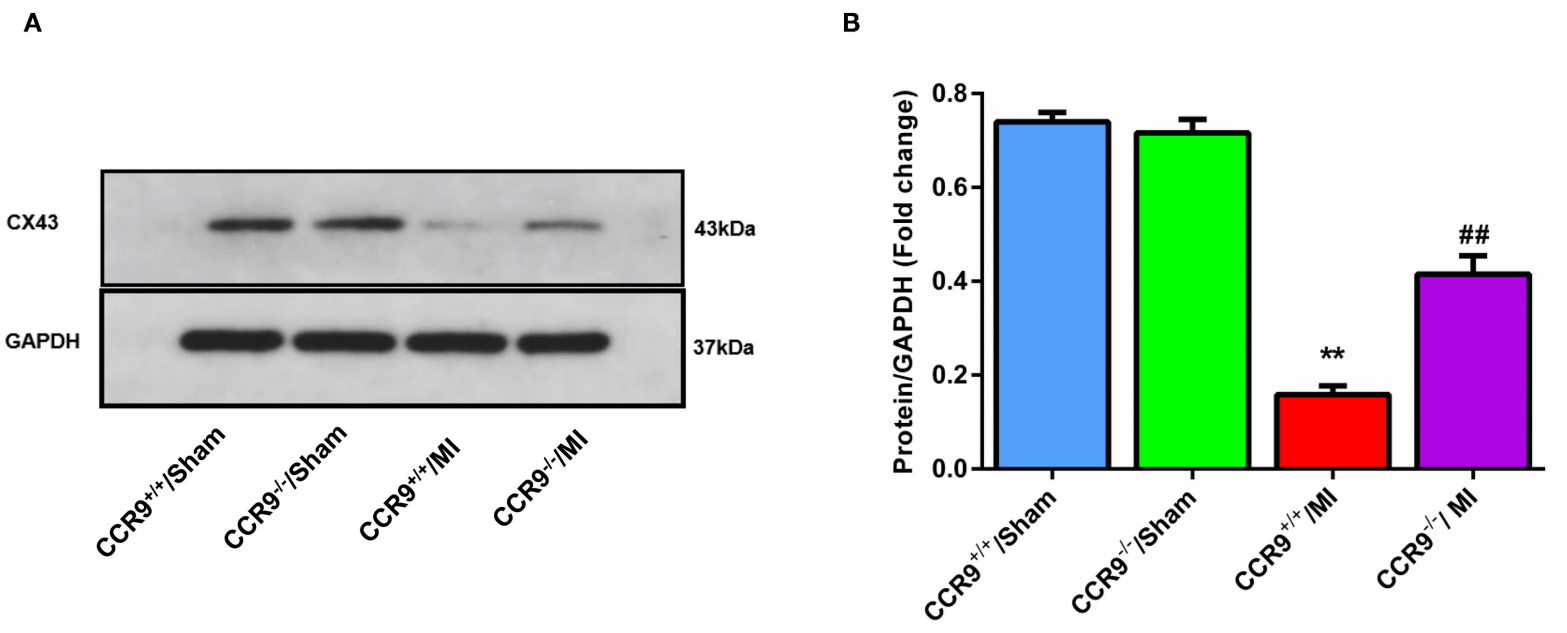
CCR9 knock out preserves the expression of Cx43. **(A)** Western blot band of Cx43 in sham or MI mice heart tissues. **(B)** Cx43 expression level in each group (**p < 0.01 vs. CCR9^+/+^/Sham, ^##^*p* < 0.01 vs. CCR9^+/+^/MI).

The westernblot result revealed that the expression level of Cx43 was decreased following MI (Protein/GAPDH, 0.16 ± 0.02 in CCR9^+/+^/MI group, *n* = 4 hearts vs. 0.74 ± 0.02 in CCR9^+/+^/sham group, *n* = 4 hearts, *p* < 0.01; 0.42 ± 0.04 in CCR9^−/−^/MI group, *n* = 4 hearts vs. 0.72 ± 0.03 in CCR9^−/−^/Sham group, *n* = 4 hearts, *p* < 0.01), while loss of CCR9 could preserve the expression of Cx43 (Protein/GAPDH, 0.42 ± 0.04 in CCR9^−/−^/MI group, *n* = 4 hearts vs. 0.16 ± 0.02 in CCR9^+/+^/MI group, *n* = 4 hearts, *p* < 0.01, Figures 5A,B).

## Discussion

The major findings in this study include: (1) The APD was prolonged, *I*_*Ca,L*_ was increased and *I*_*to*_ was decreased following MI in mice, while CCR9 knockout attenuated these changes. (2) CCR9 knockout reversed the calcium transient and calcium content reduction in sarcoplasmic reticulum following MI. (3) The conduction function was preserved in CCR9^−/−^/MI mice due to the preserved Cx43 expression.

SCD is the main cause of death in patients with acute MI. Arrhythmia usually occurs in the early phase of acute MI, and ventricular arrhythmia is the most common cause of death in MI patients (4). The main factors of arrhythmia after MI include: first, the heterogeneity of scar repair is the risk matrix for arrhythmia after MI, and the more severe myocardial fibrosis, the higher the incidence of arrhythmia (23–25). After MI, collagen fibers were deposited in the surrounding area of infarction, and the surviving myocardial cells and collagen fibers interacted with each other. Due to the electrical heterogeneity between myocardial cells and fibroblasts, reentry produced and electrical conductivity became abnormal, which induce arrhythmia after MI (26–28). Studies have shown that when myocardial cells were co-cultured with fibroblasts, the higher the proportion of fibroblasts, the slower the conduction speed, and the higher incidence of abnormal electrical activity (29). Through *in vivo* study, Michael and colleagues reported that Cx43 expression at myofibroblast-cardiomyocyte junctions was much less than that in remote region, which may result in low electrical conductivity in myofibroblast-cardiomyocyte junction area (30). Secondly, inflammatory response after MI can promote the occurrence of arrhythmia. Inflammatory response can cause both structural and electrical remodeling. A negative inflammatory factor can activate fibroblasts to differentiate into fibroblasts, increase collagen synthesis and fibrosis, and thus induce arrhythmia. On the other hand, cytokines and other inflammatory factors can directly affect the function of ion channels, affect proteins that regulate intracellular calcium homeostasis and intercellular gap junction (31–34).

**Figure 6:**
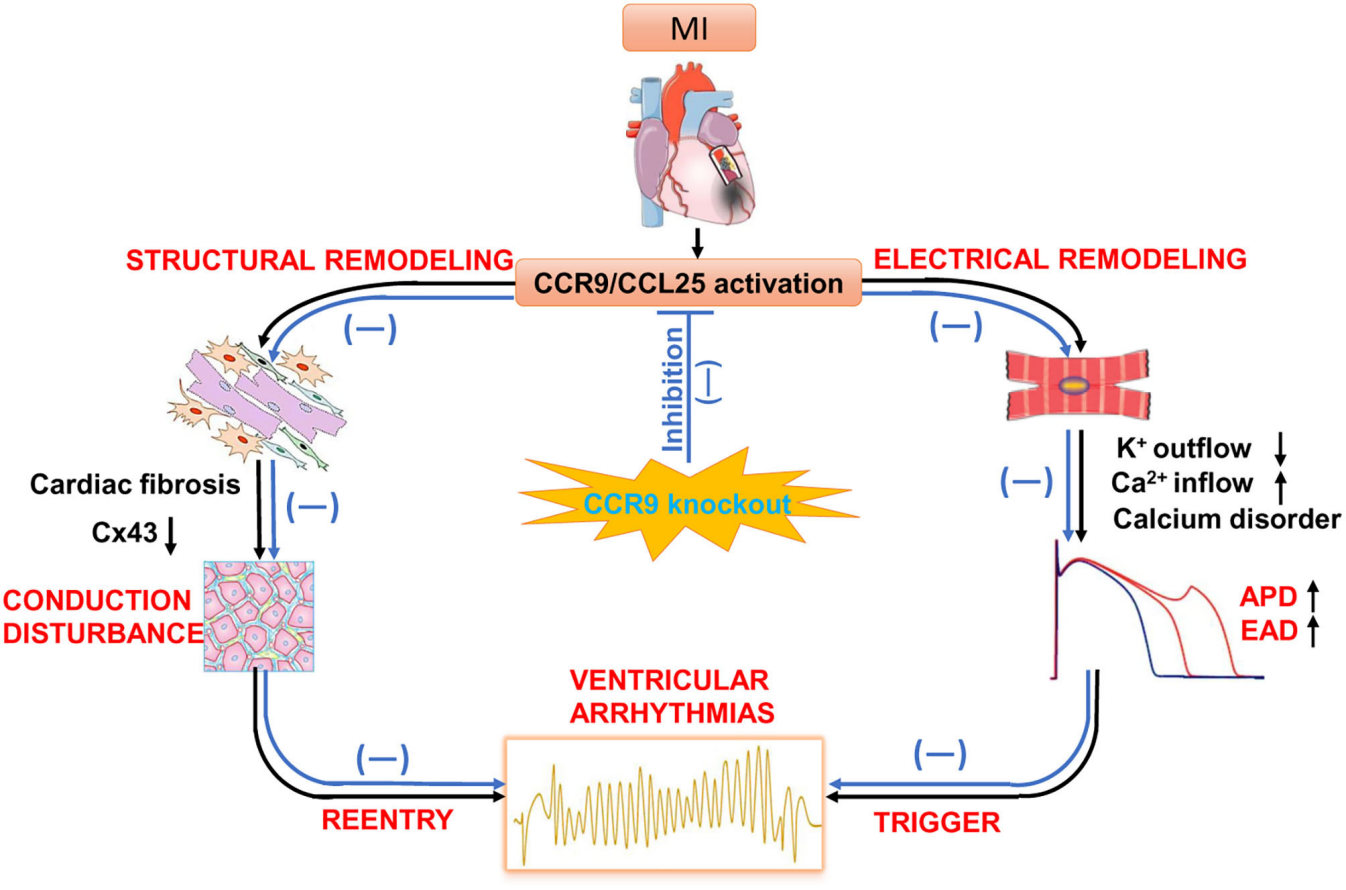
The potential role of CCR9/CCL25 in cardiac remodeling after MI. Following MI, CCR9/CCL25 is activated. Inflammatory cells tend to distribute toward the infarct area and secrete inflammatory factors. Cytokines accelerate structural and electrical remodeling by activating cardiac fibrosis, impairing gap junction *via* changes in connexin 43 expression, disrupting calcium homeostasis. These pathological changes increase ectopic activity, decrease ventricular conduction and promote reentry, which are substrate for ventricular arrhythmias. Whereas, CCR9 knockout improves cardiac inflammation and fibrosis by inhibiting CCR9/CCL25 activation, as well as inflammatory cytokines chemotaxis, which maintains expression of connexin43 and electrical activity of cardiomyocytes, and preventing ventricular arrhythmias.

Our previous investigation found that severe inflammatory response and myocardial fibrosis occurred after myocardial infarction, and CCR9 gene knockout can alleviate inflammation and myocardial fibrosis. Therefore, we attempted to further explore whether CCR9 gene knockout also has an impact on electrical remodeling after MI. In this part of the study, CCR9 gene knockout mice were studied to establish an acute MI model, and the cell electrophysiological properties were explored. We found that after MI, the APD_90_ of the ventricular myocytes in the remote area was prolonged, which can be explained by the increased *I*_*Ca,L*_ and the decreased *I*_*to*_. These results were accordant to other studies on electrophysiological consequences following MI (35). However, for CCR9^−/−^/MI mice, the APD_90_ was shortened compared with wild type mice after MI. Consistent with APD_90_ alternation, the *I*_*Ca,L*_ was decreased and *I*_*to*_ was also increased in CCR9^−/−^/MI mice when compared with wild type MI mice. This finding is novel. CCR9 knockout improves cardiac inflammation and fibrosis by inhibiting CCR9/CCL25 activation as well as inflammatory cytokines chemotaxis, which maintains electrical activity of cardiomyocytes and reduces the APD prolongation as well as the ion current disorder (Figure 6).

Also, we showed that the electrical conductivity was slowed and gap junction protein expression of Cx43 was decreased obviously after MI, which was consistent with the results reported in literatures (36, 37), which have shown that Cx43 knockout mice under myocardial ischemia stress is prone to occur ventricular tachycardia. Cardiac conduction block and anisotropy happened in conditional Cx43 knockout mice, and ventricular arrhythmia susceptibility increase (38–40). Cx43 is the main protein that constitutes the gap junction between myocardial cells. Gap junction mediate movement of ions from cell to cell and is vital for impulse conduction through the Purkinje fibers and ventricular myocardium (41). After MI, the expression of Cx43 is reduced, and the uncoupling between myocardial cells can cause the electrical conduction block between myocardial cells, and subsequently induce malignant arrhythmia. Thus, inflammation, fibrosis, abnormal ion channel function as well as decreased Cx43 expression after MI triggering conduction abnormality, contribute to the reentry. Meanwhile, calcium handling disorder accelerates the occurrence of triggered activity. Triggering and reentry are the pathological basis of arrhythmia and sudden cardiac death (Figure 6).

Our further study previously showed that CCR9 gene knockout can reduce inflammation and improve the structural remodeling after MI. The results of our current study indicated that CCR9 gene knockout can shorten the prolonged APD after MI through influencing the calcium current and potassium current, as well as the calcium handling, which indicated that inhibiting CCR9 can improve the electrical remodeling and reduce the occurrence of malignant arrhythmia after MI. Moreover, CCR9 gene knockout reserves the conduction function and the expression of Cx43 in myocardial tissue after MI, suggesting that CCR9 knockout diminishes the inflammatory response and fibrosis, which can consequently save some Cx43 expression from MI induced degradation, and thus alleviated the electrical uncoupling and conduction abnormalities among myocardial cells. At the same time, CCR9 gene knockout reduces inflammatory response and fibrosis, and these structural remodeling also play an important role in improving electrical remodeling. Therefore, early inhibition of CCR9 expression or chemotaxis of CCR9 positive cells in acute MI is expected to prevent malignant arrhythmia after MI.

## Limitations

There are limitations to be addressed for the present study. First, this study is based on mice, which has different pathophysiological procedures from human beings. So, further exploration on larger animals as well as patients is warranted. Secondly, only CCR9 knockout mice are used here. It would be optimal if transgenic CCR9 mice could also be applied as another study group. Additionally, we need to perform more study to explore the underlying mechanisms and pathway in the future.

## Conclusion

To address arrhythmias following MI, drugs and implantable cardioverter defibrillators (ICDs) as well as radiofrequency ablation were applied, while the mortality is still high and there are side effects for drugs and life quality is reduced due to devices implanted. Therefore, novel treatment strategies including gene therapy are in urgent need. In this study, CCR9 abrogation was suggested to ameliorate MI-induced electrical remodeling by affecting ion current, AP, calcium homeostasis and cardiac conduction as well as gap junction, suggesting it may be a novel pharmaceutical target for the treatment of MI-induced arrhythmia.

## Data Availability Statement

The raw data supporting the conclusions of this article will be made available by the authors, without undue reservation.

## Ethics Statement

The animal study was reviewed and approved by the Animal Care and Use Committee of Renmin Hospital of Wuhan University.

## Author Contributions

YH, DH, C-XH, and H-SD, conceived and designed the study and drafted the manuscript. YH, DH, H-SD, TS, TW, Y-HT, and Y-TC performed the experiments. YH, DH, H-SD, Y-TC, and HB-M analyzed the experiment data. YH, C-XH, and DH wrote the manuscript. All co-authors participate in editing of the manuscript.

## Funding

This research was supported by the National Natural Science Foundation Project of China (Grant No. 81670304 and 8210020476) and the Fundamental Research Funds for the Central Universities of China (No. 2042019kf0058).

## Conflict of Interest

The authors declare that the research was conducted in the absence of any commercial or financial relationships that could be construed as a potential conflict of interest.

## Publisher's Note

All claims expressed in this article are solely those of the authors and do not necessarily represent those of their affiliated organizations, or those of the publisher, the editors and the reviewers. Any product that may be evaluated in this article, or claim that may be made by its manufacturer, is not guaranteed or endorsed by the publisher.
